# Extreme High Prevalence of a Defective Mannose-Binding Lectin (*MBL2*) Genotype in Native South American West Andean Populations

**DOI:** 10.1371/journal.pone.0108943

**Published:** 2014-10-14

**Authors:** José Raul Sandoval, Hans O. Madsen, Gianfranco De Stefano, Jaime Descailleaux-Dulanto, Margarita Velazquez-Reinoso, Cesar Ñique, Ricardo Fujita, Peter Garred

**Affiliations:** 1 Centro de Genética y Biología Molecular (CGBM), Facultad de Medicina Humana, Universidad San Martín de Porres, Lima, Perú; 2 Laboratory of Molecular Medicine, Department of Clinical Immunology, Section 7631, Rigshospitalet, University of Copenhagen, Copenhagen, Denmark; 3 Department of Biology, Universita Tor Vergata, Rome, Italy; 4 Laboratorio de Genética Humana, Facultad de Biología, Universidad Nacional Mayor de San Marcos, Lima, Perú; 5 Universidad Catolica Santo Toribio de Mogrovejo, Facultad de Medicina Humana, Lambayeque, Perú; Oxford University, United Kingdom

## Abstract

Mannose-binding lectin (MBL) is one of the five recognition molecules in the lectin complement pathway. Common variant alleles in the promoter and structural regions of the human MBL gene (*MBL2*) influence the stability and serum concentration of the protein. Epidemiological studies have shown that *MBL2* variant alleles are associated with susceptibility to and the course of different types of infectious and inflammatory conditions. However, it has been suggested that these alleles are maintained in different populations due to selected advantages for carriers. We investigated the *MBL2* allelic variation in indigenous individuals from 12 different West Central South America localities spanning from the desert coast, high altitude Andean plates and the Amazon tropical forest within the territories of Peru (n = 249) (Departments of Loreto, Ucayali, Lambayeque, Junin, Ayacucho, Huancayo and Puno), and Ecuador (n = 182) (Region of Esmeraldas and Santo Domingo de los Colorados). The distribution of *MBL2* genotypes among the populations showed that the defective variant *LYPB* haplotype was very common. It showed the highest frequencies in Puno (Taquile (0.80), Amantani (0.80) and Anapia (0.58) islander communities of the Lake Titicaca), but lower frequencies of 0.22 in Junin (Central Andean highland) and Ucayali (Central Amazonian forest), as well as 0.27 and 0.24 in the Congoma and Cayapa/Chachis populations in the Amazonian forest in Ecuador were also observed. Our results suggest that the high prevalence of the *MBL2 LYPB* variant causing low levels of functional MBL in serum may mainly reflect a random distribution due to a population bottleneck in the founder populations.

## Introduction

Mannose-binding lectin (MBL) is a pattern recognition molecule that recognizes sugars such as terminal mannose and N-acetyl-glucosamine groups common on the surface of various microorganisms [1]. MBL plays a role in innate immune defence by mediating activation of the lectin complement pathway via three associated serine proteases (MASP-1, MASP-2 and MASP-3, respectively) and by enhancing phagocytosis of microorganisms and dying host cells [2]. It shares features with other recognition molecules in the lectin complement pathway, ficolin-1, ficolin-2 and ficolin-3 [3] and collectin-11 [4].

The *MBL* gene is located at chromosome 10 (10q11.2-q21). In lower primates and in mammals there are 2 functional MBL genes while in higher primates and in humans there is only one functional MBL gene (*MBL2*) [5]. MBL is primarily expressed and synthesized by the liver and subsequently released to the blood stream. Exon 1 encodes the signal peptide, a cysteine rich domain and seven copies of a repeated Glycine-Xaa-Yaa motif typical for the triple helix formation of collagen structures (Xaa and Yaa indicate any amino acid). This pattern is continued by additional 12 Glycine-Xaa-Yaa repeats in exon 2. Exon 3 encodes a neck region and exon 4 a carbohydrate-binding domain. The resulting protein consists of oligomers each with 3 identical polypeptide chains of 25 kD [6]. When isolated from serum the protein consists of 3 to 6 identical oligomers.

In the general population the protein has been shown to be of particular importance in protection against bacterial and viral infections during the vulnerable period of infancy between 6 and 18 months of age prior to establishment of specific immune protection provided by the adaptive immune system [7]. In addition, it has been ample documented that low levels of functional MBL serum are associated with increased risk of different types of infections in patients with an accompanying disease or immunodeficiency [8], [9]. By contrast it has also been shown that high levels MBL might be disadvantage in relation to certain inflammatory conditions [10].

The genetic basis of reduced serum MBL concentration has been explained at the molecular level by the identification of 3 missense variant alleles in exon 1, differentially distributed according to ethno-geographical location [11]. The common normal allele is called *A* and the structural variants are called *B* (Gly54Asp) (rs1800450); *C* (Gly57Glu) (rs1800451) and *D* (Arg52Cys) (rs5030737) respectively. These variants are dominant in the Mendelian sense and the mutations are located at the Cys- and Gly- collagen-like domain regions and even in heterozygotes the levels of functional serum MBL are diminished 10 times because the allele hampers the oligomerization of the protein, which affects the interaction with the MASPs and the opsonization processes [6]. Three additional promoter single nucleotide polymorphisms (SNPs) (G to C) that affect serum MBL levels have been detected at position –550, (named H and L) (rs11003125), at position –221 (named X and Y) (rs7096206), and in the 5′UTR at position +4 (named P and Q) (rs7095891). These 3 SNP locations together with exon 1 variants were grouped as four-marker haplotypes and 7 of them have been detected in different human populations: 4 structurally normal but differentially expressed haplotypes *HYPA*>*LYQA*>*LYPA*>*LXP*A and 3 structural variant haplotypes *LYPB*, *LYQC* and *HYPD* [12].

The relatively high prevalence of defective low *MBL2* alleles (*B*, *C* or *D*) in most populations studied and their association with infectious diseases has led to the hypothesis that the presence of these alleles represent a balanced genetic system with selective advantages for heterozygotes [13]. Preliminary studies in a few indigenous populations showed frequencies as high as 0.46 for allele B at the Southern Cone of South America [12].

To get insight to the distribution of the *MBL2* alleles and haplotypes in different indigenous people of South America we have investigated populations situated in different regions at the Pacific desert coast, high altitude Andean plates and the Amazonian forest within the territories of the Countries of Peru and Ecuador.

## Results

### *MBL2* genotype and MBL serum oligomerization pattern correlation

**Figure 1:**
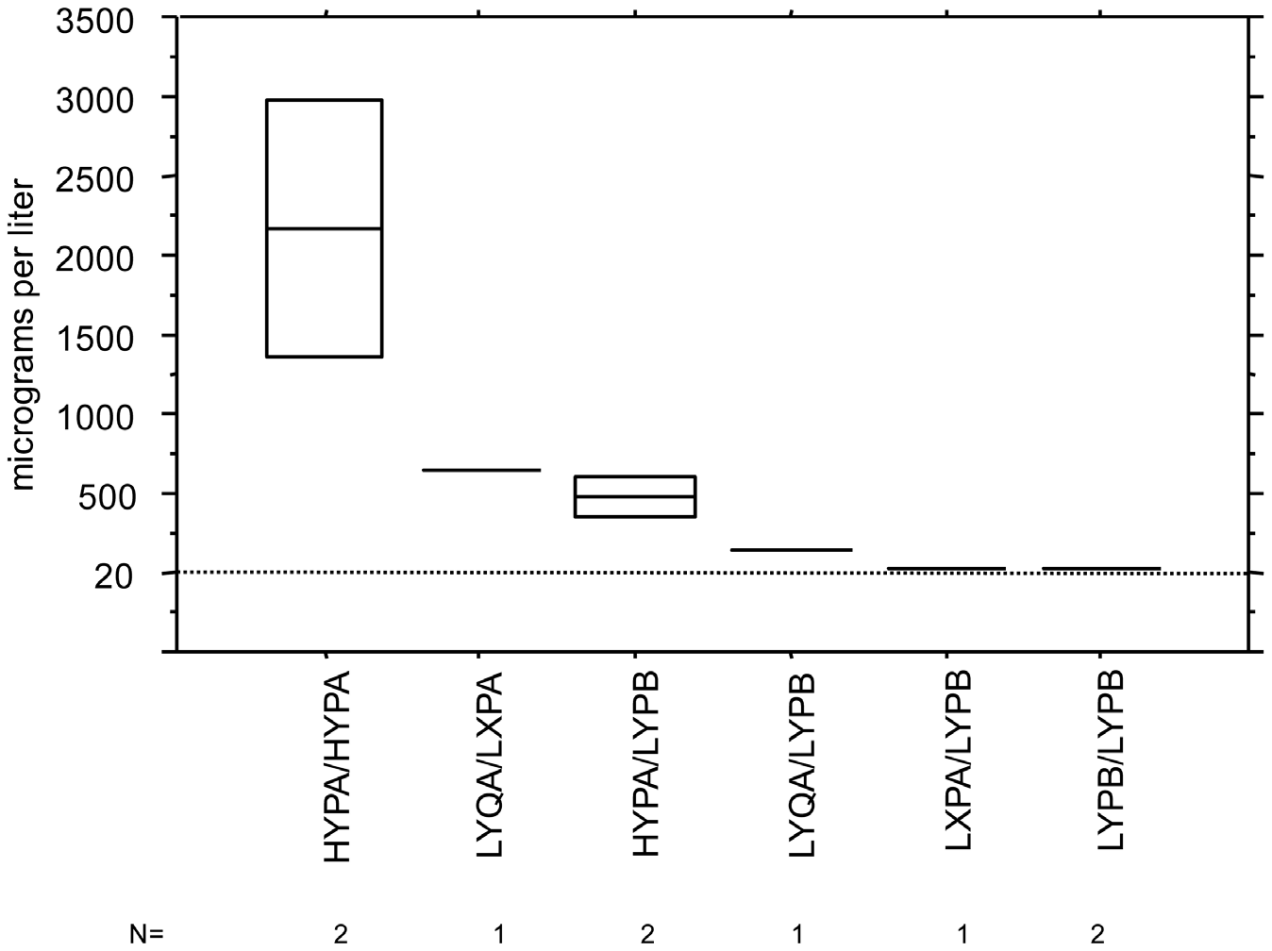
Mannose-binding lectin serum concentrations correlated with MBL2 promoter and structural genotypes in 7 native Peruvians. Horisontal line indicates either actual concentration or mean from 2 individuals.

**Figure 2:**
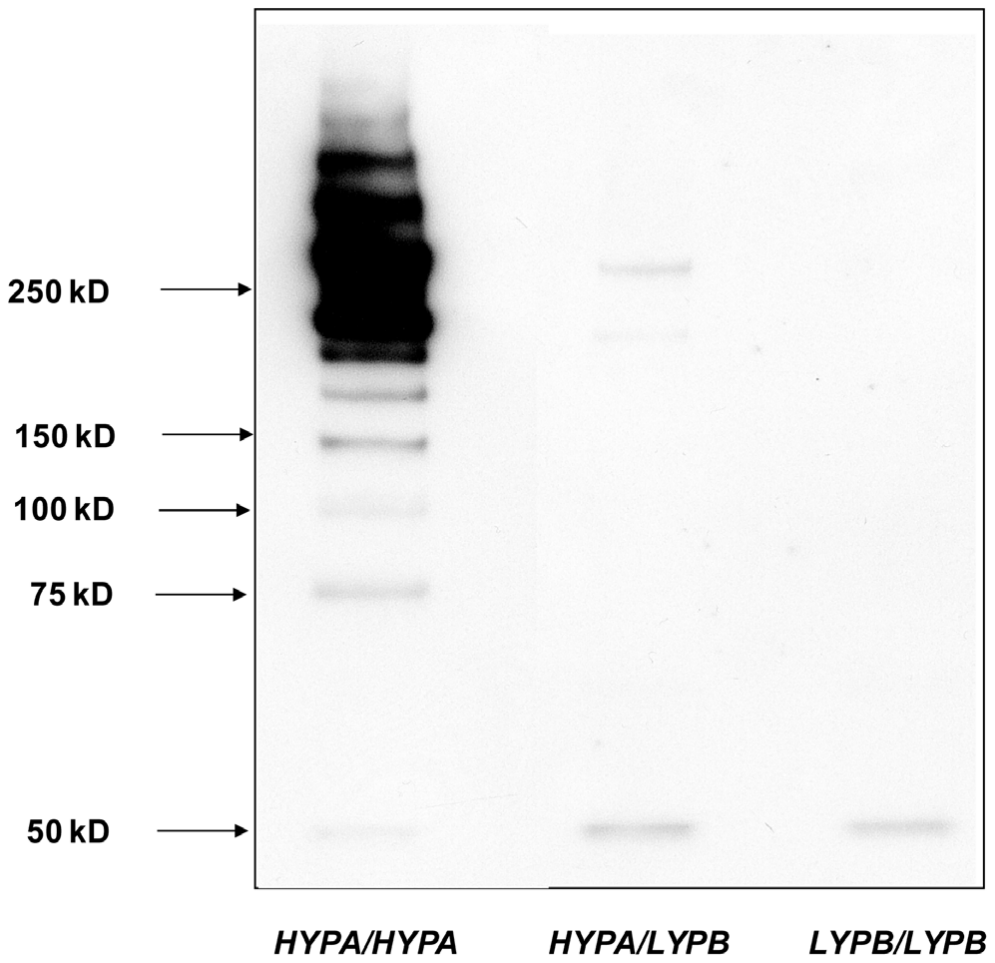
Variations in Mannose-Binding Lectin Oligomerization according to the *MBL2* haplotypes *HYPA/HYPA*, *HYPA/LYPB* and *LYPB/LYPB* from 3 different individuals.

The level of MBL serum concentrations in the tested Peruvian samples according to the accompanying genotypes are shown in Figure 1. In Figure 2 is the oligomerization pattern of MBL according to different MBL genotypes shown.

### Haplotypes

In the investigated Peruvian and Ecuadorian populations we found 7 globally reported haplotypes. No new *MBL2* haplotypes were detected. The exact test for observed and expected heterozygosity did not show any deviation from the Hardy–Weinberg equilibrium model (HWE) among all studied populations (p-value>0.05).

### Variant LYPB are common in South Native American populations with very high frequencies in islanders of the Lake Titicaca

Examination of *MBL2* haplotypes revealed that the *LYPB* was common in all of the studied populations, but that the variants *LYQC* and *HYPD* were virtually absent (Table 1). The lowest frequencies of the *LYPB* haplotype ranged from 0.22 (0.10 homozygous) in the Peruvian departments of Junin (Central Andean highland) and Ucayali (Central Amazonian forest), as well as 0.27 (0.03 homozygous) and 0.24 (0.05) respectively in the Congoma and Cayapa/Chachis population at the Amazonian forest in Ecuador. The highest frequencies were recorded at the islands of the Lake Titicaca (3800 m over the sea level in the Andes), Taquile and Amantani with a frequency of 0.80 (0.64 of homozygous), and also at Anapia with a frequency of 0.58.

**Table 1:** Frequencies of *MBL2* haplotypes and variant homozygous individuals in different populations (P = Peru, E = Ecuador).

Populations	*HYPA*	*LYPA*	*LYQA*	*LXPA*	*LYPB Gly54Asp*	*LYQC Gly57Glu*	*HYPD Arg52Cys*	Variant homozygotes
Kenya* (n = 61)	0.08	0.13	0.25	0.24	0.02	0.24	0.04	0.13
Mozambique* (n = 154)	0.06	0.30	0.27	0.13	-	0.24	-	0.06
Caucasian* (n = 250)	0.31	0.04	0.19	0.26	0.11	0.03	0.06	0.03
Eskimo* (n = 72)	0.81	0.04	0.01	0.03	0.12	-	-	0.03
Mapuche* (n = 25)	0.38	0.08	-	0.04	0.46	0.04	-	0.16
Chiriguano* (n = 43)	0.54	0.02	0.01	0.01	0.42	-	-	0.14
Iquitos-P (n = 30)	0.65	-	0.05	0.033	0.27	-	-	0.16
Pucallpa-P (n = 20)	0.55	0.08	0.10	0.05	0.22	-	-	0.10
Huancayo-P (n = 20)	0.63	0.13	-	0.02	0.22	-	-	0.10
Ayacucho-P (n = 40)	0.49	0.10	0.01	0.05	0.35	-	-	0.10
Taquile-P (n = 30)	0.18	0.02	-	-	0.80	-	-	0.64
Amantani-P (n = 30)	0.18	0.02	-	-	0.80	-	-	0.63
Anapia-P (n = 19)	0.39	0.03	-	-	0.58	-	-	0.31
Los Uros-P (n = 26)	0.63	0.02	-	-	0.35	-	-	0.11
Lambayeque-P (n = 34)	0.53	0.03	-	0.01	0.41	0.01	-	0.21
Chiguilpe-E (n = 36)	0.49	0.06	0.04	0.04	0.36	0.01	-	0.19
Cóngoma –E (n = 39)	0.72	-	-	0.01	0.27	-	-	0.03
Cayapas-E (n = 107)	0.72	0.03	-	-	0.24	-	0.01	0.05

* Madsen *et al.*, 1998.

### Native American populations bear mainly haplotypes *HYPA* and *LYPB*

It is noticeable that all the American subpopulations analyzed here and in previous studies showed that the haplotypes *HYPA* and *LYPB* are the most prevalent. Both Eskimos at the Northern, and Mapuche and Chiriguanos at the Southern extremes of the Americas have shown these haplotypes as the most represented in their population [12]. The present study performed in more meridional populations indicated that these haplotypes are also prevalent in groups living in different ecological surrounding (i.e. warm desert, cold high altitudes and in tropical rain forest climates).

### Genetic variability and differentiation among the populations

**Figure 3:**
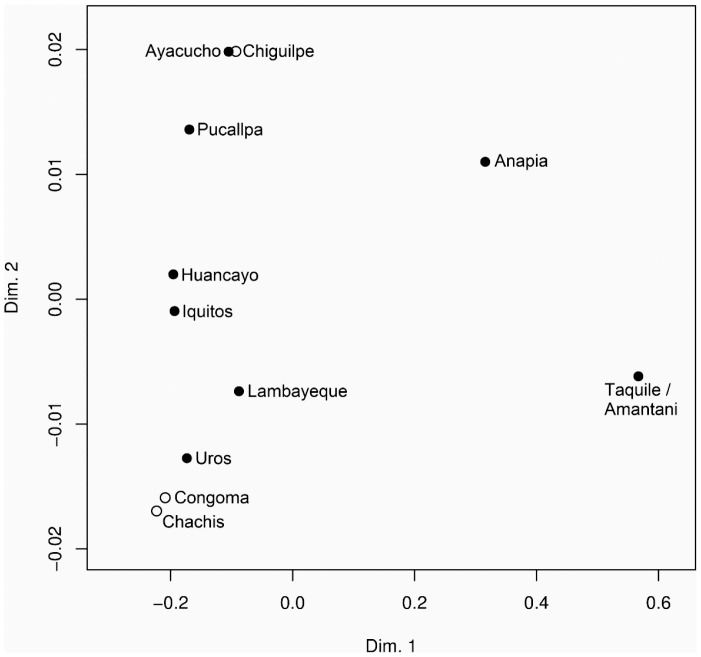
nmMDS among the Peruvian and Ecuadorian populations (stress = 0.011) using Reynolds Fst genetic distances. In black circles, Peruvian subpopulations; in white-Ecuadorian subpopulations. In the bidimensional space the Taquile/Amantani population showed that they are distant from other communities due to high frequency of the defective *MBL2* haplotype *LYPB*.

Analysis of Molecular Variance (AMOVA) was performed from *MBL2* haplotype frequencies among the Peruvian and Ecuadorian populations and it showed a moderate variation between them (Fst = 0.127; P<0,00001). The population pairwise *Φst* analyses showed statistically significant differentiation (Table 2, numbers below the diagonal) between Taquile/Amantani and other populations (p<0.05) which is visualized by the non-metric MDS plot of Reynolds genetic distances (Table 2) and numbers above the diagonal in Figure 3.

**Table 2:** Matrix of Reynolds Fst genetic distances (above the diagonal) and their Fst p-values (below the diagonal; significance level = 0.05) between the studied populations (Cha = Chachis, Cong = Congoma, Chig = Chiguilpe, Lby = Lambayeque, Aya = Ayacucho, Hyo = Huancayo, Iq = Iquitos, Puc = Pucallpa, Amt = Amantani, Taq = Taquile, Ur = Uros, Ap = Anapia).

	Cha	Cong	Chig	Lby	Aya	Hyo	Iq	Puc	Amt	Taq	Ur	Ap
Cha	-	0	0.07679	0.06988	0.07157	0.00023	0.00166	0.03829	0.58519	0.58519	0.01103	0.22902
Cong	0.71458	-	0.0528	0.04687	0.04962	0	0	0.02499	0.55761	0.55761	0	0.18808
Chig	0.00129	0.01594	-	0	0	0.02054	0.01828	0.00344	0.25403	0.25403	0.00893	0.03123
Lby	0.00475	0.03010	0.64875	-	0	0.02684	0.02175	0.02241	0.25536	0.25536	0	0.02166
Aya	0.00119	0.01218	0.90654	0.45293	-	0.01568	0.0183	0.00578	0.27693	0.27693	0.00776	0.04001
Hyo	0.20602	0.20453	0.12979	0.09603	0.19859	-	0	0	0.52908	0.52908	0	0.1527
Iq	0.30710	0.49540	0.10237	0.09900	0.07277	0.36729	-	0	0.4672	0.4672	0	0.1337
Puc	0.05396	0.08336	0.33521	0.12078	0.30185	0.59796	0.42461	-	0.43858	0.43858	0.01127	0.11816
Amt	0	0	0	0	0	0	0	0	-	0	0.41428	0.09542
Taq	0	0	0	0	0	0	0	0	0.99990	-	0.41428	0.09542
Ur	0.18959	0.37947	0.22414	0.34482	0.16543	0.29799	0.47966	0.20948	0	0	-	0.08967
Ap	0.00010	0.00119	0.08930	0.15058	0.05574	0.00485	0.00257	0.00861	0.01960	0.02188	0.02871	-

## Discussion

MBL is a recognition molecule present in serum of importance for first line host defence. It was the first recognition molecule to be discovered in the lectin complement pathway, which now has turned out to comprise at least 4 other molecules with distinct and overlapping functions [4], [14].

A striking observation with regard to *MBL2* is the presence of three missense variant alleles causing low levels and dysfunctional folding of MBL that are clustered in exon 1. Our analyses of the MBL serum concentration and the MBL oligomerization pattern in native Americans corroborate this notion (figures 1 and 2). The *B* allele is frequent in Native Americans as shown by the presence of up to 12% in Greenland Eskimos, 42% in Chiriguano and 46% in Mapuche ethnic groups, respectively in the Southern part of the Andes within Argentina territory [12]. In Eurasian populations the *B* allele is found with frequencies between 11% and 25% [15], [16]. By contrast the *B* allele is virtually absent in Sub-Saharan populations where the *C* allele is predominant reaching its highest frequencies in West-Africa (30%) [17]. The *D* allele is less frequent than the *B* and *C* alleles and has been found in Caucasian and North-East African populations with an allele frequency of 4–6%, respectively [18].

To explain this high prevalence in some populations a possible advantageous effect of the variant alleles has been proposed, suggesting that they have been evolutionary kept by its capability to avoid certain infections or diminish the effect of complement driven inflammatory reactions. However, this assumption has mounted conflicting results when tested in different populations and is still a matter of debate [15], [19]–[21].

We corroborated that the *B* allele was present at very high frequencies in all the South American populations analyzed and propose that this pattern is extended to most populations of the continent, as already shown in Southern Argentina populations and Eskimos [12]. It is noticeable that in some populations the allele *B* was the most prevalent compared with the “normal” *A* allele, reaching allele frequencies up to 80% and homozygosity frequencies of around 64% in the islands of Taquile and Amantani in the Lake Titicaca.

We find that several South American populations have higher frequencies of a defective allele of *MBL2* than any other population from other continent registered so far. After the observation of a major prevalence of this variant defective allele it is tempting to propose a still unknown protective effect is being exerted in these islands as well as the island of Anapia and in the Mapuche population with frequencies of *LYPB* reaching 0.58 and 0.46 respectively. However, it is remarkable that the island “Los Uros”, which is geographically very close to Taquile and Amantani, the *LYPB* haplotype has a frequency of only 0.35, which is difficult to conceal with the hypothesis of a specific selective effect. Thus, our hypothesis regarding “Los Uros” and the groups of Taquile, Amantani and Anapia is that the difference is caused by a genetic founder effect instead of a genetic advantage.

Historical and anthropological evidence suggest that Taquile and Amantani were repopulated after the Spanish conquerors expulsed the original populations and imposed new settlers in these islands in mid XVI century [22]. Our previous results with other genetic markers indicate that Taquile and Amantani populations are genetically very homogeneous probably originated from a reduced number of settlers [23]. Using the AMOVA test suggests that Quechua and Aymara speaking individuals are moderately differentiated (*Fst* = 0.18, p<0.01). In addition we have some evidence with mtDNA and microsatellite polymorphic markers that the Los Uros community is at least partially genetically differentiated in relation to their neighbours [24]. However, it is still possible that ancestral life style could promote differences in their genetic distribution. Taquile, Amantani and Anapia are land islands dedicated mostly to agriculture; whereas the population of “Los Uros” mainly live in artificial hay-made islands where the microenvironment, including pathogens, could be different.

It is conceivable that the microbial challenge that met the first settlers first in North America and then subsequently at the Central and South American continent thousands of years ago may have selected different alleles to become prevalent combined with bottleneck effects. It is known that there are at least 2 events in South America history showing bottleneck effects, first, the peopling the Americas about 18000 years ago by a small effective population size (estimated between 70 to 5000) [25]. And more recently around 500 years ago, in coincidence with European colonization [26]. The dramatic influence exerted by the Spanish invasion may have affected the autochthonous population dynamics. Among the most important reasons were warfare, new agriculture and ungulated farming practices, abusive colonial ruling; and probably the main cause: infectious diseases [27], [28]. The native American population shrunk dramatically after the Spanish conquest, for example in the Inca population (including modern Peru and Ecuador territories) was estimated around 17 million individuals, and this number was reduced to a little more than 1 million, one hundred years after the colonial invasion [27]. Some authors claim that the immunogenetic constitution of autochthonous Americans were so different that some Old World's diseases ravaged when passed to the Americas (*virgin soil epidemics*) [29]. Although several infectious diseases were known in the Americas like Chagas, leishmaniasis, Carrion's disease; it is considered that smallpox, measles, typhus, malaria and other “new” infectious diseases were more lethal than the firearms of the Spanish conquistadors [30].

The South American populations studied here and probably most Native American populations, have a very high prevalence of defective variant *B* allele being the most prevalent in some groups. It is also interesting to corroborate that all the previous and present Native American population studied have a prevalence of both *LYPB* and *HYPA* and the presumed intermediary LYPA haplotype is rather scarce. Based on our study it is most likely that this reflects a random distribution due to population bottleneck effects in the founder population. But it cannot be excluded that part of the observed distribution could be due to a selection against specific pathogens found among the ancestors of the autochthonous Americans or due to specific exposures before, during and after the Spanish invasion. In any case, the observed genetic pattern suggest that this co-prevalence of *HYPA* and *LYPB* was built in the Asian migrants before the passage to the American continent. A comparative analysis of Pre Columbian ancient DNA and modern indigenous populations would help to solve that enigma.

## Materials and Methods

### Population studied

A total of 429 individuals of allegedly autochthonous American and admixed populations from different climatic and altitudinal regions of Peru and Ecuador were investigated. The majority of samples were collected in relatively isolated villages of the native participants, who were interviewed in order to assess the birthplace and ethnicity of their parents and grandparents, and to certify that at least three preceding generations of their ancestors had been living in the same locality. Relatives to the 3rd degree were avoided. Samples were collected from individuals living in different regions of Peru: Departments of Loreto (Northern Amazonian forest, n = 30 from Iquitos), Ucayali (Central Amazonian forest, n = 20 from Pucallpa), Lambayeque (Northern desert coast, n = 34), Junin and Ayacucho (Central Andes, 3,600 mts above sea level, cold weather, n = 20 from Huancayo and n = 40 from Ayacucho) and Puno (islanders of the Lake Titicaca, Southeast Andes at 3880 mts above sea level, cold weather, n = 105). Samples from the Department of Puno were taken from 2 islands speaking Quechua (Taquile, n = 30 and Amantani, n = 30) and from 2 islands speaking Aymara (Isla Los Uros, n = 26 and Anapia, n = 19). We also collected 182 samples from the region of Esmeraldas (Río Cayapa) and Santo Domingo de los Colorados in the Amazonian forest of the Republic of Ecuador. Five ml of blood was drawn to obtain DNA using standard protocols from CGBM (USMP) and Department of Biology (Universita Tor Vergata) laboratories in the few cases where also serum was obtained which were isolated and snap frozen otherwise DNA samples were extracted from buccal swabs using standard procedures.

Sampling was done after verbal and written informed consent. The consent has been recorded in data sheets for each participant. The approvals of the project and the information procedure as well as ethical approval were given by Centro de Genética y Biología Molecular (CGBM), Facultad de Medicina Humana, Universidad San Martín de Porres, Lima, Peru, local ethical committee and institutional review board.

### Determination of *MBL2* alleles by sequence specific priming PCR

*MBL2* single SNPs in the form of the structural variants named *B* (codon 54), *C* (codon 57), and *D* (codon 52) as well as the regulatory variants named *H/L* (−550), *X/Y* (−221), and *P/Q* (+4) were typed by PCR using sequence specific priming (PCR-SSP), which includes 12 reactions according to the method described in [8]. As internal positive control we included a PCR covering exon 4 of the *MBL2* gene. The PCRs were performed essentially as previously described [31], except that the concentration of dNTPs was reduced to 0.7 mM, and the PCR products were analysed by a 2% agarose gel electrophoresis. Although the typing was performed as SNP-typing the results were combined in haplotypes, based on strong linkage disequilibrium between the SNPs that gives the seven known haplotypes: Four functional haplotypes *LXPA*, *LYPA*, *LYQA*, and *HYPA* (the common allele is designated “*A*”), and three “novo variants” haplotypes; *LYPB*, *LYQC*, and *HYPD* [12]. Pilot assays to optimize annealing conditions at the Lima laboratory were performed using 7 control samples representing each of the 7 known haplotypes mentioned above.

Products of the PCR amplification were individually loaded in a 2% agarose gel, stained with ethidium bromide, visualized over a UV transilluminator and recorded in a photodocumentation system. MBL serum concentrations and SDS-PAGE (NuPAGE 3–8%) and western blotting of 7 and 3 sera, respectively from genotyped native Peruvians were performed following standardized protocols [32]–[34].

### Statistical analysis

HWE and the exact test using Markov chain was performed for *MBL2* genotypes. Haplotype frequencies were also obtained by direct counting and tests of population structure and differentiation using Arlequin 3.5 software [35]. To estimate the differentiation intra- and inter- populations we use the analysis AMOVA, where Fst indices were obtained to evaluate the genetic differentiation of the 12 communities. We used genetic distances linearized with population divergence times, converting *Φst* distances into Reynolds' coancestry coefficients in Arlequin, which were used in non-metric MDS analyses (nmMDS) calculated with PAST software (http://folk.uio.no/ohammer/past) [24].
